# ASSESSMENT OF DEFICITS IN SPECIFIC COGNITIVE DOMAINS IN OLDER ADULTS LIVING WITH HIV (OALWH)

**DOI:** 10.1093/geroni/igad104.3185

**Published:** 2023-12-21

**Authors:** Andrea Reyes-Vega, Harideep Samanapally, Rishikesh Rijal, Stephen Furmanek, Christopher B Shields, Brandon C Dennis, Smita Ghare, Shirish Barve

**Affiliations:** University of Louisville, Louisville, Kentucky, United States; University of Louisville, Louisville, Kentucky, United States; University of Louisville, Louisville, Kentucky, United States; Norton Infectious Diseases Institute, Louisville, Kentucky, United States; Norton Neuroscience Institute, Louisville, Kentucky, United States; Norton Neuroscience Institute, Louisville, Kentucky, United States; Norton Neuroscience Institute, Louisville, Kentucky, United States; Norton Neuroscience Institute, Louisville, Kentucky, United States

## Abstract

A significant proportion of people living with HIV (PLWH) have cognitive impairment. Moreover, approximately 70% of PLWH in the United States will be ≥50 years old by 2030, raising concerns of a higher incidence of dementia as they age. Accordingly, there is a clinical need to monitor their cognitive status. The aim of this study was to delineate specific cognition areas impacted in OALWH with a clinical diagnosis of neurocognitive impairment. We used a comprehensive set of tests (paper and NIH Toolbox Cognition Battery), to assess different cognitive domains in a total of 25 OALWH ≥ 50 years. 64% were diagnosed with neurocognitive impairment and 36% were non-impaired. T-scores were compared using t-tests of means. Differences in means and 95% confidence intervals (CI) were reported. Impaired patients scored on average 18.35 T-score points lower on Hopkins Verbal Learning Test (HVLT) retention trial (p 0.016, CI:6.74-29.97) and 9.19 T-score points lower on the NIH Picture Vocabulary Test (PVT) (p 0.033, CI:1.12-17.26). Stroop color word, NIH Card Sort and NIH Picture sequence memory test were trending to be significantly lower in impaired patients (p< 0.07). In impaired OALWH, the HVLT data demonstrated a decreased capacity to learn and an early memory loss, suggesting frontal executive and attention type deficits. Moreover, the decreased PVT scores demonstrated an impact on crystallized intelligence, indicating decline in verbal skills, semantic knowledge, or retrieval. Overall, the observed deficits in different cognitive domains support early neurocognitive screening even when OALWH do not show overt signs of neurocognitive impairment.

